# Assessment of the Association of Health with the Liberalisation of Trade in Services under the World Trade Organisation

**DOI:** 10.1371/journal.pone.0102385

**Published:** 2014-07-31

**Authors:** Román Umaña-Peña, Álvaro Franco-Giraldo, Carlos Álvarez-Dardet Díaz, María Teresa Ruíz-Cantero, Diana Gil-González, Ildefonso Hernández-Aguado

**Affiliations:** 1 Observatory of Public Policies and Health, University of Alicante, Alicante, Spain; 2 National School of Public Health, University of Antioquia, Medellin, Colombia; 3 Public Health Research Unit, University of Alicante, Alicante, Spain; 4 Research Committee of the University of Antioquia (CODI): Research Group on public policy and health, Sustainability Strategy CODI 2013–2014, Medellin, Colombia; 5 CIBER Epidemiology and Public Health (CIBERESP), Madrid, Spain; 6 Department of Public Health, History of Science and Gynaecology, Miguel Hernández University, San Juan de Alicante, Spain; Universidad Veracruzana, Mexico

## Abstract

**Background:**

The liberalisation of trade in services which began in 1995 under the General Agreement on Trade in Services (GATS) of the World Trade Organisation (WTO) has generated arguments for and against its potential health effects. Our goal was to explore the relationship between the liberalisation of services under the GATS and three health indicators - life expectancy (LE), under-5 mortality (U5M) and maternal mortality (MM) - since the WTO was established.

**Methods and Findings:**

This was a cross-sectional ecological study that explored the association in 2010 and 1995 between liberalisation and health (LE, U5M and MM), and between liberalisation and progress in health in the period 1995–2010, considering variables related to economic and social policies such as per capita income (GDP pc), public expenditure on health (PEH), and income inequality (Gini index). The units of observation and analysis were WTO member countries with data available for 2010 (n = 116), 1995 (n = 114) and 1995–2010 (n = 114). We conducted bivariate and multivariate linear regression analyses adjusted for GDP pc, Gini and PEH. Increased global liberalisation in services under the WTO was associated with better health in 2010 (U5M: −0.358 p<0.001; MM: −0.338 p = 0.001; LE: 0.247 p = 0.008) and in 1995, after adjusting for economic and social policy variables. For the period 1995–2010, progress in health was associated with income equality, PEH and per capita income. No association was found with global liberalisation in services.

**Conclusions:**

The favourable association in 2010 between health and liberalisation in services under the WTO seems to reflect a pre-WTO association observed in the 1995 data. However, this liberalisation did not appear as a factor associated with progress in health during 1995–2010. Income equality, health expenditure and per capita income were more powerful determinants of the health of populations.

## Introduction

The process of removing obstacles to cross-border trade in goods and services known as international trade liberalisation was promoted under the assumption that free trade would ensure political stability and promote investment and job creation, thus contributing to economic growth and improving the health conditions of populations [1]. These arguments formed the rationale for the introduction of trade liberalisation as the hegemonic economic model, a model which has been promoted and often imposed by international financial institutions [2]. Established in 1995, the World Trade Organisation (WTO) is responsible for promoting and managing the multilateral liberalisation of international trade in goods and services by negotiating binding commitments among its member countries, which currently number 157 (three quarters of which are developing countries) and account for over 97% of world trade in goods and services [3].

Multilateral liberalisation of international trade in the service sector is governed by the legal framework of the General Agreement on Trade in Services (GATS) of the WTO. This sector accounts for over two-thirds of global GDP, a third of employment and about 20 percent of trade [3], [4]. Sub-sectors of the service sector include hospital services, health and social services, dental care, education, finance and telecommunications, from a total of 12 sub-sectors established by the WTO (Table 1) [5]. This variety of activities encompassed within the GATS has generated uncertainty about the potential positive or adverse effects on health. It has been argued that liberalisation exerts both a direct effect, through its impact on health systems and policies, and an indirect one, through its impact on the economic performance of countries and therefore on the living conditions of their populations [6].

**Table 1:** Service Sector Sub-Sectors as classified by the World Trade Organisation.

1	Business services
2	Communication services
3	Construction and related engineering services
4	Distribution services
5	Educational services
6	Environmental services
7	Financial services
8	Health-related and social services
9	Tourism and travel-related services
10	Recreational, cultural and sporting services
11	Transport services
12	Other services not included elsewhere

**Source:** Taken from reference 5.

The direct effects of liberalisation of international trade in the health services sub-sector are said to include attracting Foreign Direct Investment (FDI) [7], [8], promoting technology transfer, competition and efficiency, and increasing the volume and variety of services [1]. However, there is also uncertainty about its potential impact on the accessibility, equity and quality of health and social services. Market logic could prompt private hospitals to prioritise regions with more lucrative markets, to the detriment of remote areas and disadvantaged groups [1].

As for the indirect effect of liberalisation on health through its impact on the economic performance of countries, the WTO promotes liberalisation based on the premise that its capacity to attract Foreign Direct Investment (FDI) favours economic growth and poverty reduction [3]. However, the available studies on the relationship between the liberalisation of the goods sector - which has received more research attention than the liberalisation of services - and economic growth have not established a simple and unambiguous association between these two processes. Furthermore, they have produced mixed results: some have established a beneficial relationship between liberalisation, growth and therefore health [9], [10], whilst others have qualified this assertion, indicating that such benefits have been observed in a limited number of countries, most of which are located in Asia [11].

Despite these conflicting results, international financial organisations have promoted liberalisation as the dominant economic model, and therefore it is to be expected that a favourable relationship will exist between liberalisation and greater progress towards better levels of health. Thus, liberalisation would join other factors known to affect health such as wealth [12], income inequality [13] and public expenditure on health. The aim of this study was to contribute further evidence to this debate by determining the association between the liberalisation of trade in services implemented under the WTO and better levels and progress in three basic health indicators, namely life expectancy (LE), Under-5 mortality (U5M) and maternal mortality (MM) in WTO member countries, considering variables associated with social and economic policies that have been related to health, such as per capita income (GDP pc), public expenditure on health (PEH), per capita gross national income (GNI pc) and income inequality.

## Methods

### Study Design

This was a cross-sectional ecological study that explored the association between indicators of health and the service sector formal liberalisation initiated in 1995 by the creation of WTO. The indicators analysed for the dependent variables were mortality in children under five years of age (U5M), maternal mortality (MM) and life expectancy (LE). We studied the association between health and both service sector liberalisation as a whole (global liberalisation) and liberalisation limited to the sub-sector of health and social services. It was examined the association of the liberalisation’s indicators of 1995 (only data available) with health’s indicators of 2010 and 1995, and with health progress’s indicators over the period 1995–2010.

A sample was selected from the 153 WTO member countries in 2010 [14] whereby each country was used as a unit of observation and analysis. Their distribution by continent according to the United Nations [15] and by per capita income according to the World Bank [16] are shown in (Table 2). Included in the study were countries on which data were available for the indicators analysed and with 5 or more years of liberalisation, leading to a total number of countries analysed as follows: 2010 (n = 116), 1995 (n = 114), and progress over the period 1995 to 2010 (n = 114).

**Table 2:** Frequencies of WTO member countries in 2010 included in the analyses. According to region and GNI per capita.

	GNI Low		GNI Medium Low		GNI Medium High		GNI High		Total	
	2010	1995	2010	1995	2010	1995	2010	1995	2010	1995
**Africa**	20	25	13	7	5	3	-	-	38 (32%)	35 (31%)
**Asia**	4	11	8	5	6	1	4	2	22 (19%)	19 (17%)
**Europe**	-	1	1	8	5	5	23	16	29 (25%)	30 (26%)
**Latin America**	1	3	8	13	13	8	1	-	23 (20%)	24 (21%)
**North America**	-	-	-	-	-	-	2	2	2 (2%)	2 (2%)
**Oceania**	-	-	1	2	-	-	1	2	2 (2%)	4 (3%)
**Total**	25 (21%)	40 (35%)	31 (27%)	35 (31%)	29 (25%)	17 (15%)	31 (27%)	22 (19%)	116	114

**Sources:** Classification by Gross National Income (GNI) per capita according to the World Bank [16], classification by region according to the United Nations [15] and by year of WTO membership [14].

### Variables and information sources

The liberalisation indicators used were developed by the World Bank [17], both for the health and social services sub-sector, encompassing various activities established by the WTO (Table 3) [18], and for the global service sector, covering 12 sub-sectors also established by the WTO (Table 1) [5]. Each country is given a single value calculated for each of these liberalisation indicators, corresponding to the existing liberalisation commitments they have entered into in order to accede to the WTO. These indicators are scored on a scale of 0–100 according to the country’s degree of liberalisation in each of the 12 service sub-sectors established by the WTO, and the total of these. A value of zero indicates no commitments in this sector or sub-sector (minimal liberalisation), while a value of 100 indicates unlimited commitments (maximum liberalisation) [19]. Other data obtained from the World Bank included the GDP per capita (GDP pc), public expenditure on health as a percentage of GDP (PEH % GDP) [20] and Gross National Income per capita (GNI pc), which classifies countries into four categories according to their income (Low, Lower middle, Upper middle and High) [16]. The index of family income inequality (Gini) was obtained from the United Nations [21]. Information on mortality in children under 5 years of age, life expectancy and maternal mortality was obtained from the World Health Organisation [22] (Table 4).

**Table 3:** Activities encompassed within the health and social services sub-sector as classified by the World Trade Organisation.

**Hospital services**	Services delivered under the direction of medical doctors chiefly to in-patients, aimed at curing, reactivating and/or maintaining the health status of a patient. Hospital services comprise medical and paramedical services, nursing, laboratory and technical services including radiology and anaesthesiology, etc.	
**Other human** **health services**	**Ambulance services**	General and specialised medical services delivered in the ambulance.
	**Residential health facility services** **other than hospital services**	Combined accommodation and medical services not carried out under the supervision of a medical doctor located on the premises.
	**Other human health services**	Services in the field of: morphological or chemical pathology, bacteriology, virology, immunology, etc., and services not classified elsewhere, such as blood collection services.
**Social services**	**Social services with accommodation**	Welfare services delivered through residential institutions to the elderly and the disabled
		Welfare services delivered through residential institutions to children and other clients
	**Social services without accommodation**	Child day-care services including day-care services for the disabled
		Guidance and counselling services n.e.c. related to children
		Welfare services not delivered through residential institutions
		Vocational rehabilitation services

**Source:** Taken from reference 18.

**Table 4:** Definition of the study variables.

**Liberalisation under the General Agreement on Trade in Services (GATS) of the World Trade Organisation (WTO):** Indicators developed by the World Bank. These yield a value on a scale of 0–100, depending on the level of liberalisation commitments entered into by each country, for each of the 12 service sub-sectors established by the WTO, and for the total of these. A value of zero indicates no commitments in this sector or sub-sector (minimal liberalisation), while a value of 100 indicates unlimited commitments (maximum liberalisation). In the analyses, we used two indicators of liberalisation developed by the World Bank: 1) GATS commitments restrictiveness index - all service sectors: estimates the level of liberalisation in the service sector as a simple average of the 12 component sub-sectors established by the WTO. 2) GATS commitments restrictiveness index - health/social services: estimates the level of liberalisation in the of social and health services sub-sector.
**Maternal mortality ratio per 100,000 live births:** The maternal mortality ratio (MMR) is the annual number of female deaths from any cause related to or aggravated by pregnancy or its management (excluding accidental or incidental causes) during pregnancy and childbirth or within 42 days of termination of pregnancy, irrespective of the duration and site of the pregnancy, for a specified year (expressed per 100,000 live births).
**Mortality rate, under-5 (per 1,000):** The under-five mortality rate is the probability per 1,000 that a newborn baby will die before reaching age five, if subject to current age-specific mortality rates.
**Life expectancy at birth, total (years):** Life expectancy at birth indicates the number of years a newborn child would live if prevailing patterns of mortality at the time of its birth were to stay the same throughout its life.
**GDP per capita:** GDP per capita is gross domestic product divided by midyear population. GDP is the sum of gross value added by all resident producers in the economy plus any product taxes and minus any subsidies not included in the value of the products. It is calculated without making deductions for depreciation of fabricated assets or for depletion and degradation of natural resources. Data are in constant U.S. dollars.
**Gross National Income (GNI per capita):** GNI per capita is the gross national income, converted to U.S. dollars using the World Bank Atlas method, divided by the midyear population. GNI is the sum of value added by all resident producers plus any product taxes (less subsidies) not included in the valuation of output plus net receipts of primary income (compensation of employees and property income) from abroad. GNI, calculated in national currency, is usually converted to U.S. dollars at official exchange rates for comparisons across economies. Economies are divided according to 2010 GNI per capita, calculated using the World Bank Atlas method. The groups are: low income, $1,005 or less; lower middle income, $1,006–$3,975; upper middle income, $3,976–$12,275; and high income, more than $12,275.
**Health expenditure, public (% of GDP):** Public health expenditure consists of current and capital spending from government (central and local) budgets, external borrowing and grants (including donations from international agencies and non-governmental organisations), and social (or compulsory) health insurance funds.
**Gini Index:** Measures the extent to which the distribution of income (or consumption) among individuals or households within a country deviates from a perfectly equal distribution. A value of 0 represents perfect equality, a value of 100 perfect inequality.

Progress indicators were calculated for each health variable, representing the decrease in maternal and infant mortality or the increase in life expectancy for each country over the period 1995–2010. To this end we identified, from the values of 2010 of all countries, the lowest value of each mortality and the highest life expectancy value, and these were considered “attainable ideal values” at the end of the period (AivU5M2010, AivMM2010 and AivLE2010). We then calculated the “ideal potential improvement” (Ipi) of each country for each health indicator, from their values in 1995 to the Aiv in 2010 (IpiU5M = U5M1995–AivU5M2010; IpiMM = MM1995–AivMM2010, and IpiLE = AivLE2010–LE1995). We also calculated the “observed improvement” (Oi) achieved by each country between 1995 and 2010 (OiU5M = U5M1995–U5M2010; OiMM = MM1995–MM2010, and OiLE = LE2010–LE1995). Lastly, we calculated the ratio between the Oi and Ipi in each country, considering this ratio as the progress (Pgss) achieved by each country (PgssU5M = OiU5M×100/IpiU5M; PgssMM = OiMM×100/IpiMM, and PgssLE = OiLE×100/IpiLE).

### Statistical Analysis

Bivariate analyses were performed (Pearson’s correlation) between the two indicators of trade liberalisation in services (global liberalisation in the service sector and liberalisation in the health and social services sub-sector), the health indicators (U5M, MM and LE) and the socio-economic indicators selected (Gini, GDP pc and PEH % GDP). In cases of significant bilateral relationships, we subsequently performed multiple linear regression models, initially employing 2010 data, to determine the existence or not of an association between each liberalisation and health indicator, adjusting for socioeconomic indicators. Then, we performed multiple linear regressions with the liberalisation indicators using 1995 data for the other indicators (health, GDP per capita, public health expenditure and Gini), in other words, data from prior to the entry into force of liberalisation commitments, to identify any possible association at that time. Lastly, we performed linear regressions between the progress in health indicators for the period 1995–2010 and liberalisation indicators, adjusting for GDP per capita, public health expenditure and Gini for 1995, in other words, the conditions of the countries at baseline. We did not conduct multivariate analyses with the liberalisation indicator for the health and social services sub-sector because the bivariate analyses did not reveal any significant associations. Statistical analyses were performed using the Statistical Package for Social Sciences (SPSS) version 15.0.

## Results

Results of the bivariate analyses performed using 2010 data showed that countries with higher global liberalisation in the service sector had lower Under-5 mortality (−0.532; p<0.001) and maternal mortality (−0.490; p<0.001) and increased life expectancy (0.524; p<0.001). In addition, greater liberalisation was associated with higher income (0.512; p<0.001), more public expenditure on health (0.544; p<0.001) and a lower Gini index, i.e. less income inequality (−0.485; p<0.001). Meanwhile, increased global liberalisation was associated with greater progress in reducing Under-5 mortality (0.379; p<0.001) and an increase in life expectancy (0.408; p<0.001) for the period 1995–2010. No significant associations were found between liberalisation of the health and social services sub-sector and health indicators (Table 5).

**Table 5:** Correlations between indicators of liberalisation, health, macroeconomics and policies.

	Liberalisation in the Healthand Social Services sub-sector		Global liberalisation in the Service sector	
	Coefficient (p value)	n	Coefficient (p value)	n
**Under-5 mortality (U5M) 2010**	−0.136	116	−0.532	116
**Maternal mortality (MM) 2010**	−0.106	116	−0.490	116
**Life expectancy (LE) 2010**	0.104	116	0.524	116
**Under-5 mortality (U5M) 1995**	−0.076	114	−0.514	114
**Maternal mortality (MM) 1995**	−0.068	114	−0.476	114
**Life expectancy (LE) 1995**	0.067	114	0.520	114
**U5M progress 1995–2010**	0.132	114	0.379	114
**MM progress 1995–2010**	0.043	114	−0.150	114
**LE progress 1995–2010**	0.059	114	0.408	114
**GDP per capita**	0.018	116	0.512	116
**Public expenditure on health** **% GDP**	0.201	116	0.544	116
**Gini**	−0.239	116	−0.485	116

Members in 2010 with 5 or more years of liberalisation and data availability. Pearson’s Correlation.

Results of the multiple linear regression analyses performed using data from 2010 indicated that final models accounted for 35% of the variability in U5M, 29% in MM and 43% in LE. In these models their principal determinants were global liberalisation in the service sector and GDP per capita. Thus, it was observed that the higher the GDP per capita and level of liberalisation in the service sector, the lower the rates of Under-5 and maternal mortality and the higher the values for life expectancy (Table 6).

**Table 6:** Linear regression models.

	Under-5 mortality(per 1000 live births)	Maternal Mortality(per 100000 live births)	Life Expectancy (in years)
	Coefficient (p value)	Coefficient (p value)	Coefficient (p value)
**Constant**	112.828	469.768	61.360
**Global Liberalisation**	−0.358 (<0.001)*	−0.338 (0.001)*	0.247 (0.008)*
**GDP per capita**	−0.275 (<0.013)*	−0.283 (0.014)*	0.448 (<0.001)*
**Gini**	−0.082 (0.361)	−0.090 (0.339)	−0.020 (0.815)
**Public expenditure on** **health % GDP**	−0.136 (0.210)	−0.094 (0.404)	0.070 (0.485)
	n = 116/R^2^ = 0.350	n = 116/R^2^ = 0.296	n = 116/R^2^ = 0.436

Under-5 and Maternal Mortality and Life Expectancy with Global Liberalisation in the Service Sector under the WTO. 2010.

**Method:** entered in SPSS.

*p = <0.05.

Results of the regression analyses performed using data from 1995, the year in which the WTO was established, also indicated a positive association between global liberalisation of services and health indicators. These models accounted for 37%, 33% and 44% of the variability in U5M, MM and LE, and their principal determinants were global liberalisation of the service sector and government expenditure on health and GDP per capita (Table 7).

**Table 7:** Linear regression models.

	Under-5 mortality(per 1000 live births)	Maternal Mortality(per 100000 live births)	Life Expectancy (in years)
	Coefficient (p value)	Coefficient (p value)	Coefficient (p value)
**Constant**	132.969	619.927	55.753
**Global Liberalisation**	−0.249 (0.011)*	−0.221 (0.028)*	0.196 (0.033)*
**GDP per capita**	−0.154 (0.128)	−0.096 (0.354)	0.284 (0.003)*
**Gini**	−0.0002 (0.998)	−0.021 (0.817)	−0.019 (0.818)
**Public expenditure on** **health % GDP**	−0.334 (0.002)*	−0.383 (0.001)*	0.309 (0.002)*
	n = 114/R^2^ = 0.371	n = 114/R^2^ = 0.332	n = 114/R^2^ = 0.440

Under-5 and Maternal Mortality and Life Expectancy with Global Liberalisation in the Service Sector under the WTO. 1995.

**Method:** entered in SPSS.

*p = <0.05.

Results of the multiple linear regressions performed using health progress data for the period 1995–2010 indicated that the principal determinants were income inequality and wealth of the countries, whereby greater inequality in income distribution was associated with lower progress, whilst greater wealth per capita was associated with more progress. These models accounted for 20%, 19% and 50% of the variability in U5M, MM and LE progress, respectively (Table 8).

**Table 8:** Linear regression models.

	Progress in reducing Under-5 mortality	Progress in reducing Maternal Mortality	Progress in increasing Life Expectancy
	Coefficient (p value)	Coefficient (p value)	Coefficient (p value)
**Constant**	56.676	93.988	48.873
**Global Liberalisation**	0.161 (0.141)	0.048 (0.661)	−0.016 (0.852)
**GDP per capita**	0.026 (0.816)	0.326 (0.005)*	0.465 (<0.001)*
**Gini**	−0.280 (0.005)*	−0.269 (0.008)*	−0.323 (<0.001)*
**Public expenditure** **on health % GDP**	0.134 (0.253)	−0.286 (0.170)	0.084 (0.365)
	n = 114/R^2^ = 0.206	n = 114/R^2^ = 0.191	n = 114/R^2^ = 0.501

Progress in reducing Under-5 and Maternal Mortality and increasing Life Expectancy with Global liberalisation in the Service Sector under the WTO. Period 1995–2010.

**Method:** entered in SPSS.

*p = <0.05.

## Discussion

The results indicate that in 2010, higher levels of liberalisation in the service sector under the WTO were associated with better levels of health, but this association was also observed in data from 1995, the year of entry into force of the liberalisation processes analysed, and therefore these associations cannot be attributed to an effect of the WTO. Moreover, greater progress in reducing mortality and increasing life expectancy over the period 1995–2010 was associated with lower income inequality and higher GDP per capita of the countries at the beginning of the study period. Consequently, we believe that liberalisation in the service sector under the WTO is not clearly identified as a factor associated with progress in health variables. Our analysis did not detect any association between liberalisation of trade in the health and social services sub-sector and levels of health in the countries.

When interpreting the findings, it should be borne in mind that our study was limited to analysing liberalisation in the service sector and did not consider liberalisation in the goods sector. Moreover, the indicators employed referred exclusively to liberalisation assumed in compliance with the WTO, and did not take into account any unilateral, bilateral or plurilateral liberalisation commitments that the countries may have assumed, which might limit the explanatory power of the study. However, the indicators do reflect whether or not the countries are under obligation to achieve a certain level of liberalisation, and they are also the only indicators currently available with extensive sectoral coverage [23]. The aggregate nature of our study has limited analytical depth, which could be achieved with a case study. Furthermore, the use of secondary data could represent a limitation due to lack of availability for some countries and because their quality depends on the institutions responsible for collecting them. However, the information sources and methodology used in this study represent the only means to conduct a global test of the hypotheses.

Our results are not in agreement with some of the existing evidence, upheld by the World Bank, which has established a positive and unequivocal relationship between liberalisation and better health through economic growth. This premise is based on studies that analysed liberalisation in the goods sector, which has received more research attention than the service sector, and found higher rates of growth in more liberalised groups of developing countries [24]. Such growth would lead to an increase in income among poorer segments of the population, favouring access to education and health [9]. However, these studies and this causal pathway have been the subject of controversy and qualification. Some authors have argued that those liberalisation processes considered successful because they generated economic growth have been restricted to a limited number of countries which present a given set of characteristics, such as the existence of adequate physical and human infrastructures, strong regulatory institutions, and process implementation rates that diverge from those established by international financial organisations [11]. They have also maintained that some of these countries already showed considerable economic growth before implementation of their liberalisation processes [25].

Regarding the causal pathway, some cross-sectional studies found no evidence of a systematic relationship between liberalisation and growth [26], whilst others have identified a wide range of effects on growth [9], [11]. Thus, studies on the liberalisation of trade in goods have found that East Asian countries have achieved much better growth performance with lower tariff cuts than countries such as Brazil or Peru. In other cases, such as Kenya and Nicaragua, liberalisation provoked economic stagnation or decline. Thus, the association between liberalisation and economic growth is determined by a number of factors both internal and external to countries [27], which influence any potential effect on health.

Moreover, even should economic growth occur, its impact on health through providing some sectors of the population with access to food, health care and housing [28] will also be determined by the level of income distribution [27]. Recent studies have indicated that there was a weak correlation between income growth and changes in health over the last 40 years, suggesting that although important, economic growth is not essential for progress in health. Factors that could explain health gains include national health and education policies and the spread of innovations in medicine through concerted action programmes implemented by the international community [21].

The lack of an association in our results between liberalisation of the health and social services sub-sector and health indicators suggests that other factors may have more impact on the health of populations. It may also reflect the absence of an association between significant increases in investment flows in this sub-sector and commitments under the GATS [29], a partial reason for which might be that the current commitments in all sub-sectors were more a consolidation of the levels of market access in place at the time of signing in 1995, rather than an increase in liberalisation [1], [30].

If the commitments made under the WTO in 1995 were indeed a consolidation of existing liberalisation in services, then the favourable association observed in 1995 may reflect a probable health benefit of this pre-WTO liberalisation, which would persist until 2010. If this were the case, it would be reasonable to expect to find a similar association over the period 1995–2010, in which new conditions converged to favour trade in services, such as the entry into force of binding GATS commitments and the emergence of new communication technologies. However, our results for progress in health over this period did not reveal any such association. Rather, they indicated that the pattern of the WTO’s liberalisation of trade in services was not associated with progress in health between 1995 and 2010, and that factors such as unequal income distribution and the wealth of countries have been more powerful determinants. It is also clear that countries which improve their levels of health achieve greater progress in economic development [21].

The liberalisation of services under the WTO does not emerge as a factor in improving the health of populations. This coincides with a considerable proportion of the evidence that questions the thesis promoted by financial institutions, who contend that there is a strong and positive relationship between liberalisation and the economic and social development of countries. On the contrary, the relationship between the liberalisation of services and health is far from clear. Therefore, it becomes necessary to prioritise the creation of policies that favour the development and attainment of health benefits as a prerequisite for the implementation of liberalisation processes. In this regard, our results support the importance of public policies that favour the redistribution of wealth and access to public services as mechanisms to ensure equity in the health of populations.
