# Correction: Assessment of the Association of Health with the Liberalisation of Trade in Services under the World Trade Organisation

**DOI:** 10.1371/journal.pone.0113560

**Published:** 2014-11-07

**Authors:** The PLOS ONE Staff

The third author's name is spelled incorrectly. The correct name is: Carlos Álvarez-Dardet. The correct citation is: Umaña-Peña R, Franco-Giraldo Á, Álvarez-Dardet C, Ruíz-Cantero MT, Gil-González D, et al. (2014) Assessment of the Association of Health with the Liberalisation of Trade in Services under the World Trade Organisation. PLoS ONE 9(7): e102385. doi:10.1371/journal.pone.0102385
